# Construction and implement of hierarchical management system for specialist nurses based on Patricia Benner’s theory

**DOI:** 10.3389/fmed.2024.1472384

**Published:** 2024-11-21

**Authors:** Haiyan Yu, Yuan Chen, Linjing Wu, Ligang Wang, Yuting Lai, Aijun You

**Affiliations:** ^1^Xiamen Cardiovascular Hospital, Xiamen University, Xiamen, China; ^2^School of Nursing, Fujian University of Traditional Chinese Medicine, Fuzhou, China; ^3^Emergency Department, Xiamen Hospital, Zhongshan Hospital, Fudan University, Xiamen, China

**Keywords:** specialist nurse, nursing practice teaching, hierarchical, construction, system

## Abstract

**Objective:**

To construct a hierarchical management system for specialist nurses based on Patricia Benner’s theory, and evaluate its implement effect, so as to provide reference for the hierarchical management of specialist nurses.

**Methods:**

Literature retrieval, semi-structured interview and Delphi method were conducted for initially formulation of the draft of hierarchical management system for specialist nurses. Forty-three specialist nurses and 14 nursing managers were selected for the study, using a non-randomized controlled experimental study design, and at the end of the study, the job satisfaction, job engagement, job exuberance, advice behaviors and nursing managers’ overall job satisfaction of specialist nurses were compared before and after the hierarchical management.

**Results:**

This study constructed a hierarchical management system for specialist nurses. The differences in specialist nurses’ job satisfaction, job engagement, job exuberance and constructive behaviors before and after hierarchical management were statistically significant (*p* < 0.05), and the differences in the nursing managers’ assessment of specialist nurses’ overall job satisfaction were statistically significant (*p* < 0.05).

**Conclusion:**

The hierarchical management system of specialist nurses based on Patricia Benner’s theory improves the quality of hierarchical management of specialist nurses, which could improve the job satisfaction of specialist nurses. The system could provide guidance and reference for hierarchical management system of specialist nurses.

## 1 Introduction

The State of the World’s Nursing 2020 report indicated that strengthening nursing team construction and promoting high-quality development of nursing career was crucial (1). Cultivating professional nursing talent has become the key direction of nursing development in the new era (2). The emergence of specialist nurses has become a crucial driver and fundamental force in advancing the field of nursing toward high quality. Specialist nurse refers to the registered nurse who has obtained the corresponding specialist nurse qualification certificate after systematic training and examination (3); has profound nursing theoretical knowledge, expert nursing skills and rich clinical experience in a specific nursing field. Simultaneously, they actively engage in therapeutic education programs and offer support and advocacy for patients and their families (4), and they are key figures in the specialist nursing team, and their core competency level significantly impacts the quality of nursing care and the advancement of the nursing profession (5).

The National Health and Family Planning Commission of the People’s Republic of China (6) proposed to establish a hierarchical management system for specialist nurses that meet the needs of nursing positions in China by 2020. However, the management of specialist nurses faces a challenging situation that requires attention and resolution. Despite undergoing rigorous qualification training, specialist nurses have not experienced significant changes in their job positions and responsibilities in practice, leading to the widespread issue of “emphasizing training but not utilization.” Consequently, specialist nurses are unable to fully leverage their professional capabilities, which not only impacts their work motivation but also hampers their professional satisfaction. Secondly, due to the lack of unified and standardized qualification and training standards, there is a significant gap in the professional competence of specialist nurses. The gap not only affects the quality of specialist nursing care but also restricts the sustainable development of the specialist nursing career. Furthermore, the traditional nursing management model has been challenging to adjust to the requirements of the contemporary medical environment, which emphasizes task completion and quantity, overlooking the enhancement of nursing quality and patient satisfaction (7). This management approach not only hinders the professional growth of nurses but also impacts the overall efficacy of nursing services. Therefore, it is crucial to construct a hierarchical management system for specialist nurses.

Scholars have proposed a model of clinical progression for nurses at all levels of training. The hierarchical management of nurses strictly follows the principle of core competence requirements and competence level correspondence of specialist nurses to ensure that they can perform various duties according to their professional competence and post characteristics (8). This enables them to leverage their professional strengths and advantages, ultimately enhancing the overall quality and efficiency of nursing services.

Patricia Benner’s theory was developed by Dr. Benner, a professor in the Department of Physiological Nursing at the University of California, San Francisco School of Nursing. This theory serves as a framework for establishing a hierarchical management system for specialist nurses. According to Patricia Benner’s theory, it’s primarily used to assess the needs of nurses at various stages of their professional development (9). Nurses are required to progress through five stages: novice, advanced, competent, proficient, and expert (10). Specialist nurses at each stage exhibit distinct characteristics, each with its own corresponding training needs. Additionally, in related literatures use the term “nurse practitioner” to categorize specialist nurses into five level (11, 12).

Therefore, the purpose of our study was to construct a hierarchical management system and implement it for specialist nurses at various levels. This will offer scientific and theoretical references for a hierarchical management system tailored to specialist nurses.

## 2 Methods

A mixed-method research design with a quantitative component was used. The literature review, semi-structured interview, and Delphi method were employed to construct a more scientific and reliable hierarchical management system for specialist nurses.

### 2.1 Construction of a hierarchical management system for specialist nurses

#### 2.1.1 Setting up research team

**Figure 1:**
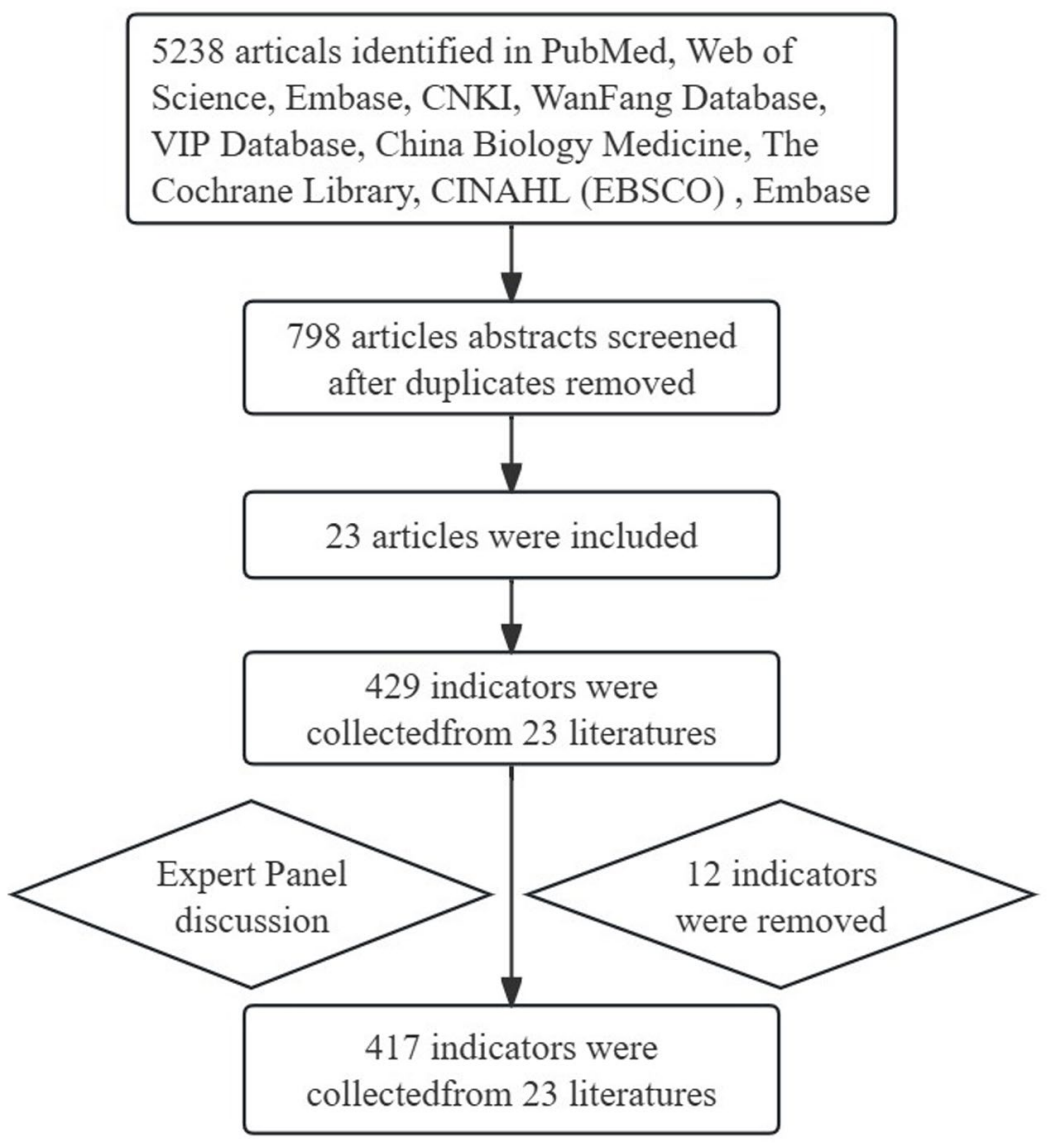
Benner’s five-stage “novice to expert” clinical competency model.

The research team was composed of 7 personnel, including a director of nursing, a clinical head nurse, a nursing department education nurse, two specialist nurses, a clinical nurse and research nurse a clinical. The director of the nursing department was responsible for coordinating and supervising the work arrangement and progress, the education nurse of the nursing department was responsible for formulating the study protocol, the clinical head nurse, specialist nurses, clinical research nurse and clinical nurse were responsible for literature review, semi-structured interviews, questionnaire development and consultation with experts (see Figure 1).

#### 2.1.2 Literature retrieval

**Figure 2:**
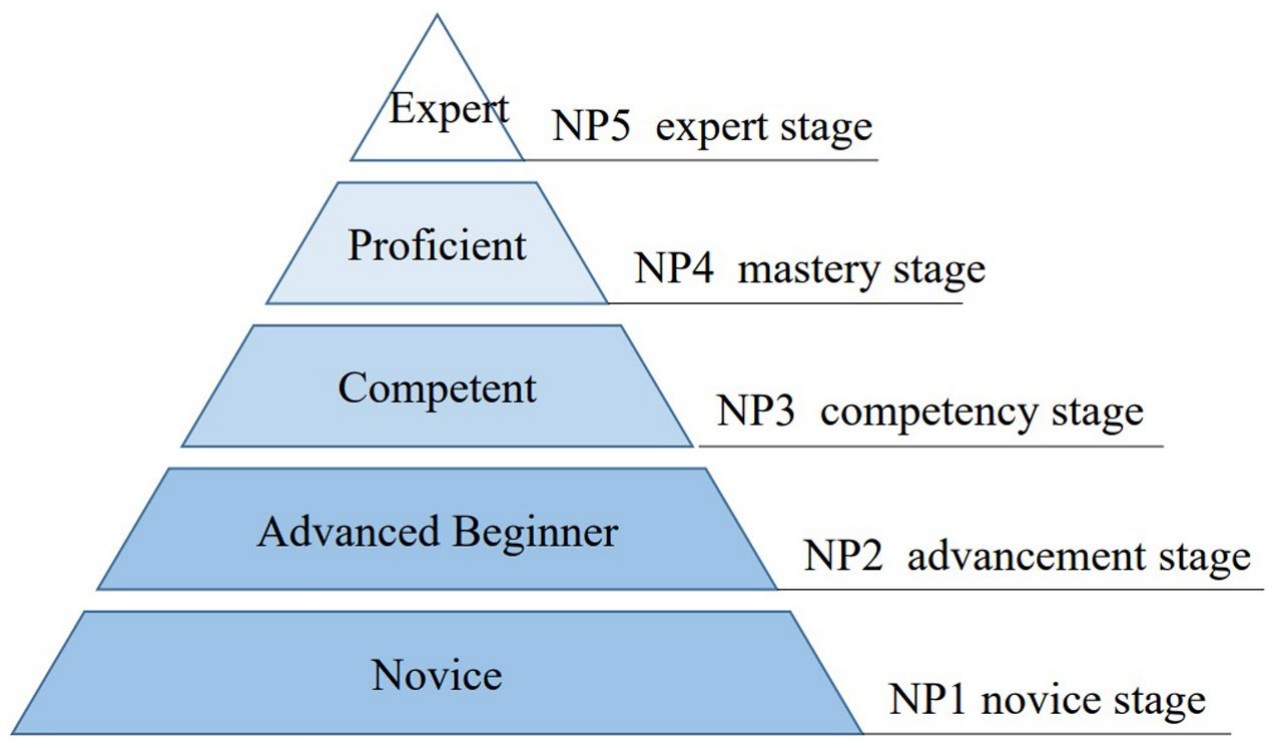
Screening process of hierarchical management system indicators.

The research group searched a large number of relevant literatures at home and abroad and traced back the references of the included literature guided by Benner’s theory. The literatures were found through search of PubMed, Web of Science, The Cochrane Library, CINAHL (EBSCO), Embase, China National Knowledge Infrastructure (CNKI), WanFang Database, VIP Database, and China Biology Medicine. The keywords included “Specialist Nurse, Clinical Nursing Expert, Advanced Practice Nurse, Advancement, and more.” Two reviewers worked independently to scan and evaluate full literature. So, 23 studies were eventually included. Initially, the first draft of the hierarchical management system for specialist nurses with 7 first-level indicators, 32 s-level indicators, and 376 third-level indicators were identified. Including job setting, access standards, initial grading standards, promotion standards, job responsibilities, continuing education, assessment standards and other content. The process of screening was displayed in Figure 2.

#### 2.1.3 Semi-structured interview

Purposive sampling method was used to select five specialist nurses and nursing managers from a tertiary hospital in Fujian Province to conduct semi-structured interviews for 30 min/person. The outline of the interviews included: ① What do you think specialist nurses are like? ② What differences do you think exist between the current working status of yourself/specialist nurses and your expectations? Why? ③ What problems do you think exist in the current utilization and management of specialist nurses? How do you hope to improve it? ④ What do you think needs to be improved in the current management of specialist nurses? The interviews were promptly transcribed, and the content analysis method was used to analyze and refine the themes of the interview data. Members of the research team revised the first draft of the hierarchical management system for specialist nurses based on the results of the interviews and removed two tertiary indicators, and finally the specialist nurses’ hierarchical management system was formulated after the collective discussion and approval of the research team members.

#### 2.1.4 Delphi method

The Delphi method involves multiple rounds of consultation by using questionnaires to obtain the collective opinion of experts until a consensus is reached (13). It usually involves 2–3 rounds of consultation, and the interval between the 2 rounds is generally 4–5 weeks. This method was applied in this study, which included drafting Delphi survey questionnaires, collecting expert feedback and establishing indicators through expert evaluation and discussion. In this study, we defined the term “expert” as someone who: (1) graduated with a bachelor’s degree or above in nursing; (2) has had at least 10 years of clinical work and 5 years of clinical education or management experience in the hospital; (3) should obtain the intermedium or advanced level certificate; (4) they should voluntarily participate in the investigation and promise to participate in two rounds of consultation. The purpose of the study and expert consultation process was explained via email. We encouraged the experts to refer other potential experts interested in the research. After screening potential experts according to the above criteria, the authors contacted them via e-mail and explained the relevant information with the survey. It is recommended that a panel consists of 15–50 members (14), we recruited 20 panel members from 3 tertiary teaching hospitals and a university in Fujian Province, China, from August to October 2021. In the second round, experts were invited to answer the revised questionnaire in the same way as round 1 to reach a consensus. A high level of consensus was achieved after round 2. Table 1 presents the expert demographic data.

**Table 1:** Expert demographic’ characteristics (*N* = 20).

Items	*N* (%)
Gender	
Female	20 (100%)
Age (year, mean ± SD)	41.17 ± 3.06
Year of working	18.33 ± 4.08
Education lever (%)	
Bachelor’s degree	13
Master’s degree	7
Vocation (%)	
Nurse	19
Teacher	1

#### 2.1.5 Quality control

In order to make sure that the research results were representative and reliable, we had proposed some basic requirements in selecting appraisal experts. And the expert-returned questionnaires were checked carefully, and incomplete questionnaires were removed. After all correspondence forms were returned, the researcher (HY) and team member (YL) read and analyzed the open-ended responses independently. These comments were discussed by the research group to modify, collapse, or exclude each item based on the study aims and literature.

After literature review, semi-structured interviews, and Delphi method, a hierarchical management system for specialist nurses was finally constructed was finally constructed as shown in Supplementary material.

### 2.2 Implementing the hierarchical management system for clinical specialist nursing staff

In this research, a non-randomized controlled experimental study design was adopted. Forty-three specialist nurses and 14 nursing managers from a hospital were selected for the study using the convenience sampling method from June 2022 to March 2024. The inclusion criteria for the specialist nurses were (1) having nursing qualifications, (2) being engaged in clinical nursing work, holding a junior title or above, (3) passing the initial appointment of NP at the hospital level, (4) obtaining informed consent, and being willing to cooperate with the survey. The inclusion criteria for the nursing managers were (1) graduated with a bachelor’s degree or above in nursing; (2) has had at least 5 years of clinical work experience in the hospital; (3) obtained the intermedium or advanced level certificate. The study was approved by the Ethics Committee. The general information of the study population is detailed in Table 2.

**Table 2:** Demographic information of research population (*N* = 57).

Item		Frequency (*N*)	Proportion (%)	Item		Frequency (*N*)	Proportion (%)
Age (year)	20–30	17	0.4	Positions	Chief Nursing Officer, Sub-Specialist Nurses Nursing Department	1	0.07
	31–40	23	0.53		Nurse manager	13	0.93
	41–50	3	0.07	Age (year)	30–35	3	0.21
Genders	Woman	37	0.86		36–40	5	0.36
	Man	6	0.14		41–45	6	0.43
Educational background	Master’s degree	1	0.02	Educational background	Master’s degree	4	0.29
	Bachelor	38	0.88		Bachelor	10	0.71
	Junior college	4	0.09	Management experience (year)	<5	7	0.5
NP	N1	14	0.33		5–10	4	0.29
	N2	14	0.33		11–15	3	0.21
	N3	15	0.35	Acquisition of Specialist Nurse Certificate	Specialist Nurse Certificate, Chinese Nursing Association	6	0.14
Working experience (year)	<1	4	0.09		Specialist Nurse Certificate for Provincial Institutions	7	0.16
	1–2	16	0.37		Certificate of Specialist Further Training	11	0.26
	3–5	16	0.37		None	19	0.44
	6–10	4	0.09				
	11–15	0	0				
	>15	3	0.07				
Positions	Chief instructor	8	0.19				
	Teaching secretary	3	0.07				
	Junior instructor	3	0.07				
	Teaching staff	10	0.23				
	Specialty/subspecialty team leaders	9	0.21				
	Deputy head of specialty/subspecialty group	3	0.07				
	None	7	0.16				

**Figure 3:**
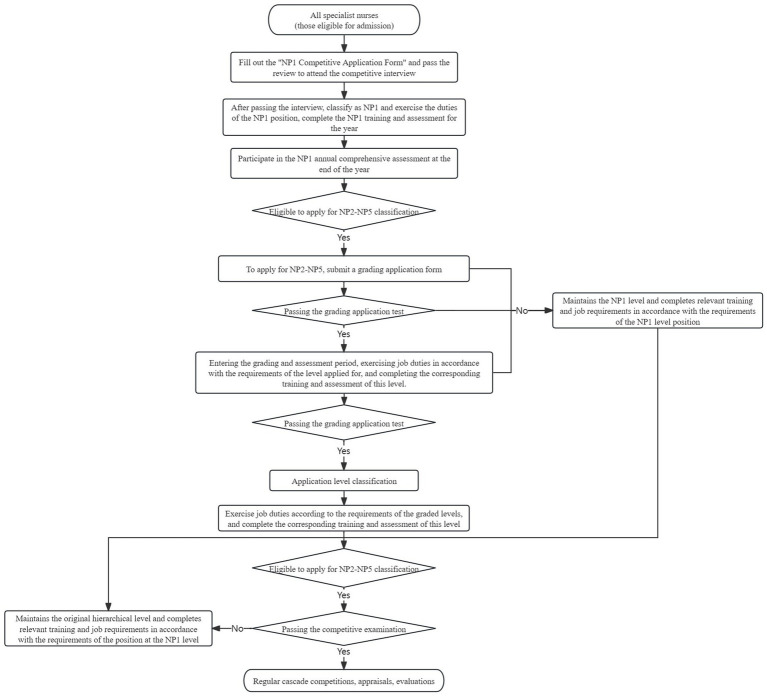
Flowchart for carrying out the management of stratified competitive recruitment of specialist nurses.

Under the coordination of the Nursing Department, we were implementing a hierarchical management system for 43 specialist nurses. Additionally, an in-hospital specialist nurse certification committee will be established, headed by the vice president for nursing. This committee is responsible for establishing and implementing an in-hospital certification system for specialist nurses to ensure that each nurse meets the established professional standards. During the accreditation process, specialist nurses who have obtained specialist nurse certificates through training by the Chinese Nursing Association or provincial institutions, as well as nurses who only possess specialist refresher certificates or meet the basic access requirements for specialist nurses in the hospital, must participate in the NP1 hierarchical competition organized by the hospital’s Specialist Nurse Accreditation Committee before being included in the specialist nurse hierarchical management. After a year of work assessment, the certification committee identified the specialist nurse hierarchical grading. This is necessary to obtain the appropriate hierarchy of specialist nurse qualification. Subsequently, training, utilization, assessment, and competition processes are determined based on the requirements of each hierarchical, as illustrated in Figure 3. At the same time, the Department of Nursing implemented the “Comprehensive Promotion of Specialty Groups Work Program” specialty group management indices, with head nurses from each department serving as specialty supervisors. They were tasked with overseeing the work of 22 specialties and 11 cardiovascular subspecialty groups across the entire hospital. Quarterly specialty group meetings were conducted, during which group members took turns presenting reports on the group’s activities and plans. The Nursing Department and specialty supervisors provided on-site guidance to assess the effectiveness of corrective actions from the previous quarter and proposed new corrective measures to ensure the smooth progress of the group’s work. Data collection on relevant evaluation indicators was synchronized during the program’s implementation.

#### 2.2.1 Outcome evaluation

##### 2.2.1.1 Minnesota Job Satisfaction Scale

The scale was developed by Weiss et al. (15), which measures and evaluates employees’ job satisfaction from three aspects: intrinsic satisfaction, extrinsic satisfaction, and general satisfaction, and contains 20 items with a total score of 0–100, with higher scores indicating higher occupational satisfaction. The Cronbach’s *α* coefficient of the scale was 0.912.

##### 2.2.1.2 Specialist Nurse Work Engagement Scale

The scale was developed by Qin et al. (16) to reflect the level of work engagement of specialist nurses in five aspects: work motivation, work attitude, work value, work recognition, work enthusiasm and concentration. Each entry is rated on a 5-point Likert scale ranging from “very much does not meet” to “the total,” the Cronbach’s *α* coefficient of the scale was 0.958.

##### 2.2.1.3 Work Exuberance Scale

This scale was developed by Porath et al. (17). It includes two dimensions: learning and vigor. Each entry is rated on a 5-point Likert scale from “strongly disagree” to “strongly agree,” with higher scores suggesting that nurses have a greater sense of job vigor. The total Cronbach’s *α* coefficient for this scale was 0.86 (18).

##### 2.2.1.4 Nurse Advice Behavior Scale

This scale was developed by Liang et al. (19). It contains two dimensions: facilitative and inhibitory speech behavior, with a total of 10 items. Each item is scored on a 5-point Likert scale, with scores ranging from 1 to 5, from “very non-compliant” to “very compliant.” Higher the score, the greater the number of nurses’ speech behavior. The scale has good reliability and validity among Chinese nurses，the Cronbach’s *α* coefficient of the scale was 0.915 (20).

##### 2.2.1.5 Nurse Satisfaction Scale

Nursing managers used the Nurse Satisfaction Scale to evaluate the overall job satisfaction of specialist nurses. The scale is based on a 7-point Likert scale, with 1 indicating very dissatisfied and 7 indicating very satisfied, and the total scale score ranges from 13 to 91. The higher the score, the greater the job satisfaction of nurses. The Cronbach’s *α* coefficient of the scale was 0.897 (21).

#### 2.2.2 Data analysis

SPSS 26.0 statistical software was utilized for data analysis. Data measurements and calculations were presented as mean ± standard deviation and frequency. The paired-sample *t*-test was employed to analyze data that followed a normal distribution, while the paired-sample rank-sum test was used for data that did not follow a normal distribution. Count data was presented as frequency and percentage and analyzed using a chi-square test. Statistical significance was considered at *p* < 0.05.

## 3 Result

Overall satisfaction of nursing managers with the tiering of specialist nurses before and after the implementation of a hierarchical management system for specialist nurses showed a statistically significant difference (*p* < 0.05). See Table 3 for details.

**Table 3:** Comparison of nursing managers’ satisfaction before and after the implementation of a hierarchical management system for specialist nurses.

	Pre-intervention	Post-intervention	*t*	*p*
Nurse Satisfaction Scale	58.07 ± 15.98	68.79 ± 14.32	−2.268^a^	0.041^*^

^*^p < 0.05.

a Paired-sample *t*-test.

Evaluation of overall satisfaction of specialist nurses before and after hierarchical management system for specialist nurses.

Overall satisfaction ratings of specialist nurses before and after stratified management, the differences in Minnesota Satisfaction Scale scores, Work Engagement Scale scores, Work Exuberance Scale scores, and Constructive Behavior Scale scores of the specialist nurses before and after hierarchical management were statistically significant (*p* < 0.05). See Table 4 for details.

**Table 4:** Comparison of specialist nurses’ satisfaction before and after the implementation of hierarchical management system for specialist nurses.

	Pre-intervention	Post-intervention	*t*	*p*
Minnesota Job Satisfaction Scale	85.05 ± 9.50	89.80 ± 10.98	-2.940^a^	0.005^*^
Specialist Nurse Work Engagement Scale	131.94 ± 13.41	137.45 ± 15.65	-2.048^a^	0.046^*^
Work Exuberance Scale	51.12 ± 8.235	56.45 ± 7.104	−3.273^a^	0.002^*^
Nurse Advice Behavior Scale	38.76 ± 5.92	41.2 ± 5.58	−2.154^a^	0.036^*^

^*^p < 0.05.

a Paired-sample *t*-test.

## 4 Discussion

National Health Commission of the People’s Republic of China proposed training 25,000 specialist nurses from 2011 to 2015 (22). After nearly 10 years of development, the team of nurse specialists has grown significantly, and the career development path of nurse specialists has been expanded. However, most medical institutions are still facing problems such as unclear job responsibilities for specialist nurses and a lack of protection in the system for the training and development of specialist nurses (23). So, it is particularly important to explore a new model for the training and development of specialist nurses. This is also the purpose and significance of our research. Therefore, based on Patricia Benner’s theory and in conjunction with the core competency requirements of specialist nurses (24), this study developed a hierarchical management system for specialist nurses and implemented it at various levels.

Based on this, our study constructed the hierarchical management system for specialist nurse staff through a variety of methods such as literature review, semi-structured interview, and Delphi method. Before the hierarchical management system contents were formulated, the research team held a group meeting to consult and listen to the opinions from nursing and education experts, and invited experts to make a feasibility assessment on the contents of the first draft of the course to ensure the practicability of the hierarchical management system. Our study constructed the hierarchical management system for specialist nurse staff through various methods, including literature review, group meetings, and Delphi method. Before formulating the course contents, the research team conducted a group meeting to consult with nursing and education experts, listened to their opinions, and invited experts to assess the feasibility of the first draft of the course content to ensure the practicality of the hierarchical management system. The finally information hierarchical management system constructed in our study included 7 indicators at the primary level, 34 at the secondary level and 376 at the tertiary level. It was complete and comprehensive for specialist nurses.

In a cross-sectional survey of more than 50,000 specialist nurses in China (25), 47.6% of respondents believed that the current lack of a systematic hierarchical management mechanism within the field of specialist nurses is an important factor that restricts specialist nurses from fully utilizing their professional strengths and potential. At present, there are indeed many different standards for the classification of specialist nurses in China. Generally, specialist nurses are categorized into three levels based on their certification: national level, provincial level, and hospital level. This model makes it difficult to establish a complete and comprehensive professional growth ladder for specialist nurses in terms of selection, training, appointment, and continuing education of specialist nurses. The growth stages of specialist nurses are similar to the growth of general nurses. They are divided into hierarchical based on the training, work, and assessment contents of specialist nurses at different stages. The hierarchical management system of nurses plays an important role in improving the professional competence and satisfaction of nurses (26).

This study was structured into five hierarchical on the specialist nurse hierarchical framework. A tiered training and assessment mechanism for in-hospital specialty nurses was gradually developed in terms of specialist nurse certification. Before specialist nurses are included in the specialist nurse hierarchical management, they are all required to participate in the NP1 hierarchical competition organized by the Intramural Specialist Nurse Certification Board. After a quarterly comprehensive assessment and an annual comprehensive evaluation of recognition, the specialist nurse hierarchical classification is determined to obtain the appropriate level of specialist nurse qualification. Subsequently, training requirements are tailored based on the different levels.

In terms of assessment standards, fair and reasonable post-assessment is an important means of incentivizing specialist nurses. Currently, there is no unified post-assessment standard or authoritative certification mechanism for specialist nurses in China (27). This study implements quarterly reporting assessments and annual comprehensive assessments for specialist nurses to ensure the continuity of their work. It formulates a system of assessment standards for specialist nurses based on specialized clinical nursing ability, specialized development ability, and comprehensive ability. Additionally, it establishes downgrading and withdrawal mechanisms for specialist nurses who fail the assessment for two consecutive years to maintain the professionalism of the specialist nurses’ team.

Addressing existing issues such as the imperfect performance appraisal system for specialist nurses and the salary-job mismatch, it is essential to establish a standardized system for appraising specialist nurses. Suggesting that the results of the comprehensive assessment of specialist nurses be used as a crucial basis for enhancing specialist nurses’ competence, job performance allocation, and title promotion; establishing a dynamic performance appraisal mechanism (28). This will lead to the establishment of a dynamic performance appraisal mechanism that fully showcases the labor value and professional worth of specialist nurses’ roles, thereby boosting their work enthusiasm. Implementing a hierarchical management system for specialist nurses, prioritizing the coordination of specialist nurses scientifically to fulfill their respective duties, offering suitable development opportunities and platforms for specialist nurses, and ensuring the quality of specialist nursing care (29). Enhancing specialist nurse satisfaction is important for stabilizing the nursing workforce and ensuring the safety of clinical care (30). The results of this study showed that the job engagement, job satisfaction, and constructive behavior scores of specialist nurses were higher after the implementation of the stratified management system for specialist nurses than before the implementation (*p* < 0.05). The implementation of hierarchical management of specialist nurses has made it possible to combine the hierarchy of specialist nurses with the job duties and technical requirements of the posts, which can effectively bring into play the core competence of specialist nurses (31). This suggests that the hierarchical management system for specialist nurses meets the requirements of specialist nurse development and promotes the improvement of specialist nurses (32). A high level of work engagement, which is a positive and complete emotional and cognitive state related to work, has a positive impact on increasing nurses’ job satisfaction and improving the quality of care (33, 34). The hierarchical management system for specialist nurses clarifies the job responsibilities at different levels, enabling specialist nurses to have a clearer understanding of their work content and processes. This helps prevent work blindness and ultimately increases the satisfaction of specialist nurses (35). At the same time, the implementation of the hierarchical management system enables specialist nurses to better define their own hierarchical planning, which is conducive to strengthening their competence and professional value. The hierarchical management system for specialist nurses provides a clear career path for specialist nurses, from NP1 to NP5, as the tier rises, the hospital will provide corresponding positions and responsibilities for specialist nurses at different tiers, and the clear positional positioning and delineation of responsibilities will help to enhance the nurses’ sense of professional development.

The results of this study show that the implementation of a hierarchical management program for nurse specialists improves the overall satisfaction of nursing managers with nurse specialists. The hierarchical management of specialist nurses involves a hierarchical framework that includes job responsibilities, promotion criteria, certification and assessment criteria. This framework clarifies the responsibilities of specialist nurses, maximizes their functions at each hierarchical, enhances the quality of specialty care (36, 37), and boosts nursing administrators’ satisfaction with specialist nurses.

## 5 Conclusion

In conclusion, we constructed a hierarchical management system for specialist nurses based on Patricia Benner’s theory and implemented it in practice. Empirical research demonstrated that the hierarchical management system significantly enhanced the specialist nurses’ hierarchical management tasks, increased nursing managers’ satisfaction with the specialist nurses’ work, and has the potential for wider adoption and implementation, which could provide reference for the hierarchical management of specialist nurses. For wider adoption, emphasizing continuous feedback loops and adaptability to evolving healthcare needs would further strengthen its impact.

This study also has some limitations. First, considering potential sources of bias or confounding factors, the extrapolation of the empirical study is limited, especially due to the use of convenience sampling method. Second, since the methods by which we evaluated effectiveness of the hierarchical management system of specialist nurses are subjective, in future research, we will conduct a high-quality multicenter randomized controlled trial and adopt a more objective method to measure the hierarchical management system of specialist nurses.

## Data Availability

The original contributions presented in the study are included in the article/Supplementary material, further inquiries can be directed to the corresponding author.
