# Supplementary material for: Construction and implement of hierarchical management system for specialist nurses based on Patricia Benner’s theory
Source: Front Med (Lausanne). 2024 Nov 21;11:1472384. doi: 10.3389/fmed.2024.1472384 (PMC11619138; doi:10.3389/fmed.2024.1472384)
Supplement: Supplementary file 1 [file Data_Sheet_1.pdf]

Hierarchical management system for specialist nurses

一、Patricia Benner’s theory

1. **NP1 (Novice stage)**：Begin to familiarize with the business of specialized nurses;
2. **NP2 (Advanced stage)**：The nurse specialist is basically proficient in her work and can provide personalized care services for patients;
3. **NP3 (Competent stage)**：Able to formulate care plans for patients based on evidence, and able to provide professional insights/opinions in the healthcare team;
4. **NP4 (Proficient stage)**：Apply evidence-based findings to the patient's situation and coordinate the multidisciplinary team to develop a care plan;
5. NP5 **(expert stage)**: able to make rapid and accurate clinical judgments and responses, with evidence-based application and research capabilities, and with influence on the healthcare team and organization.

二、Qualifications for Initial Appointment and Promotion Criteria

Admission criteria for specialized nurses: have a certificate of nursing practice; have a college degree or above in nursing and ≥5 years of clinical nursing experience; have strong business skills, communication skills, and learning ability; love and commitment to specialized nursing; and head nurses and above managers can not work concurrently.

	NP1	NP2	NP3	NP4	NP5
**Initial appointment qualifications**	1.Obtained the certificate of specialized nurse issued by provincial or above institutions or engaged in specialized nursing work recognized by the unit ≥ 1 year<br>2.College degree or above<br>3.Nurse practitioner or above title	1.Obtained the certificate of specialized nurse issued by provincial and above institutions ≥ 3 years or engaged in specialized nursing work recognized by the unit ≥ 3 years<br>2.Bachelor degree or above<br>3. Nurse or above title	1.Obtained the specialized nurse certificate issued by the provincial and above institutions ≥ 6 years<br>2.Bachelor degree or above<br>3.Supervisory nurse or above title	1.Obtaining the specialized nurse certificate issued by the provincial or above institutions ≥ 9 years<br>2.Master's degree or above/studying for Master's degree; if the specialized nurse certificate issued by provincial or above institutions is ≥15 years, the education can be relaxed to Bachelor's degree<br>3.Associate chief nurse or above title.	1.Obtained the specialized nurse certificate issued by provincial and above institutions ≥ 12 years<br>2.Master's degree or above<br>3.Associate chief nurse or above title
**Promotion Criteria**	Advancement from level “N” to level “NP1”:<br>1.the provincial and above institutions to obtain specialist nurse certificate / engaged in the unit recognized specialist nurse work ≥ 1 year<br>2.Nurse practitioner or	Advancement from “NP1” to “NP2” level:<br>1.as NP1 hierarchical specialists nurses for 2 years, during the tenure of the assessment passed<br>2.100% pass rate of NP1 level compulsory courses<br>3.Through the NP2	Advancement from “NP2” to “NP3” level:<br>1.Must obtain the specialists nurse certificate recognized by the provincial and above institutions<br>2.Have the title of nurse in charge or above<br>3.Serve as NP2 hierarchical specialists for	Advancement from “NP3” to “NP4” level:<br>1.With the title of associate chief nurse or above<br>2.as NP3 level specialized nurses for 3 years, during the tenure of assessment passes<br>3.100% pass rate of compulsory courses at NP3 level	Advancement from “NP4” to “NP5” level:<br>1.3 years as a specialized nurse at NP4 level, with passing assessment during the term of service<br>2.Passing 100% of the required courses at NP4 level<br>3.Pass the NP5 level nurse promotion examination

	above title<br>3.Tertiary education and above<br>4.Strong business skills, communication skills, learning ability, qualified in competitive assessment	hierarchical specialists nurses competitive examination, selected on the basis of merit	3 years and pass the assessment during the tenure<br>4.100% pass rate of NP2 level mandatory courses<br>5、Through the NP3 level hierarchical specialists nurse competitive examination, selected on the basis of merit	4、Through the NP4 level specialist nurse competitive examination, selected on the basis of merit	

三、Job responsibilities

The responsibilities of the specialist nurse positions at each hierarchical are based on the hamric specialist nurse core competencies (ability to provide direct clinical care, ethical decision-making, ability to provide consultation, ability to collaborate with others, ability to provide expert guidance, ability to provide leadership and management, and ability to conduct clinical research), as detailed in the table below.

core competencies	**NP1**	**NP2**	**NP3**	**NP4**	**NP5**
Ability to provide direct clinical care	1.Basic mastery of specialized nursing theory and specialized nursing skills<br>2.Can assist the superior specialized nurse to carry out simple specialized work	1.Skilled in specialized nurse job duties and processes, independently complete all shifts of specialized nursing work<br>2.Skilled in specialized nursing theory and skills<br>3.Can independently implement specialized	1.Skilled in specialized nursing duties and processes, with the ability to organize the rescue of critically ill patients<br>2.Have solid specialized nursing theory and skills, and be able to implement specialized nursing care	1.proficient in the department N3 level nurses job duties and processes, with a wealth of specialized nursing knowledge and skills, and has the ability to deal with difficult and critical specialized	1.proficient in the department N3 level nurses job duties and processes, with rich clinical nursing experience and a high level of specialized theoretical knowledge<br>2.Provide specialized

	3.Basic mastery of the duties and procedures of specialized nurses under the guidance of senior specialized nurses.	nursing care for patients with simple specialized diseases, and evaluate the effect of specialized nursing care, and appropriately dispose of nursing care.<br>4.Can carry out specialized work for patients with complex specialized diseases under the guidance of superior specialized nurses.	for patients with complex specialties.<br>3.can correctly analyze and assess the patient's condition, and according to the standard of specialized nursing care for patients to develop and implement personalized specialized nursing program, and can real-time evaluation of the effect of specialized nursing care, timely adjustment of specialized nursing programs.	nursing problems<br>2.Developing emergency response plans for specialized emergencies and assisting in dealing with social and group health care emergencies<br>3.can correctly analyze and assess the complexity of the condition, and can develop and implement personalized nursing program based on evidence, and can real-time evaluation of the effectiveness of specialized nursing care, timely adjustment of specialized nursing program<br>4.Developing the job duties and procedures of specialized nurses under the guidance of superior specialized nurses.	nursing services for the whole hospital<br>3.To assist the hospital to carry out major health rescue work or deal with the planning and implementation of health care emergencies<br>4.Able to quickly and accurately assess and analyze complex specialty difficult cases, make accurate clinical decisions, formulate personalized care plans based on the best current evidence, guide the team to provide patients with high-quality, efficient specialty care services, and continuous tracking and improvement.

**Capacity for ethical decision-making**	1.Developing specialty care plans and implementing specialty care programs within the limits of the law and in accordance with ethical principles.	1.Developing specialty care plans and implementing specialty care programs within the limits of the law and in accordance with ethical principles.	1.Developing specialty care plans and implementing specialty care programs within the limits of the law and in accordance with ethical principles.	1.Developing specialty care plans and implementing specialty care programs within the limits of the law and in accordance with ethical principles	1.Developing specialty care plans and implementing specialty care programs within the limits of the law and in accordance with ethical principles
**Provision of advisory capacity**	1.Participate in specialty difficult case nursing discussions	1.Participate in the discussion of specialized difficult cases and put forward professional insights	1.Participate in the discussion of difficult cases in the specialty, and put forward professional insights<br>2.Participate in specialized consultation	1.Preside over the specialty difficult case discussion, and put forward professional insights<br>2.Participate in the hospital specialties/ medical and nursing joint consultation	1.Professional consultant and consulting expert of the hospital's specialty care<br>2.Participate in intra- and extra-hospital specialty consultations<br>3.Chairing the discussion of specialized complex cases, and put forward professional insights
**Ability to work with others**	1.Able to coordinate and cooperate effectively with other members within the specialty group	1.Be able to raise specialty-related questions about the condition and discuss them during the room visit activities	1.Participate in the joint departmental medical and nursing room in the provision of specialty care program<br>2.In the multidisciplinary team consultation as the main reporter, and provide	1.can fully mobilize medical resources to provide patients with efficient, high-quality specialty care services, improve patient outcomes	1.Able to analyze and evaluate the multidisciplinary teamwork and medical resource utilization, and then put forward recommendations for improvement

			specialty care recommendations		2.Able to lead multidisciplinary team activities and coordinate and promote interdisciplinary teamwork.
**Expert guidance capacity**	1.Basic specialty health education for patients and their families<br>2.Regularly participate in the learning and exchange of new knowledge and skills in specialty care	1.Instructing lower level specialized nurses to carry out simple specialized nursing care<br>2.To undertake clinical basic specialty nursing operation teaching<br>3.Provide specialty education activities in the specialty team and within the department.<br>4.To undertake specialty health education for patients and their families<br>5.Participate in group health-related service activities (clinic, health consultation, etc.).<br>6.Participate in nursing academic exchange	1.To undertake the teaching of junior specialized nurses, advanced training nurses and students of specialized nurse training courses<br>2.Guide the lower level specialized nurses in the clinical practice of specialized nursing<br>3.Developing specialty health education programs, analyzing the implementation of health education and make improvements<br>4.Provide specialty education activities in specialty groups and	1.To undertake the lower level of specialized nurse training nurses and specialized nurse training course students of the teaching work<br>2.Guide the lower level specialist nurses to develop and implement the nursing care plan for difficult and critical patients.<br>3.Responsible for hospital-level specialty training programs<br>4.Guide the specialized nursing staff to carry out the evaluation of continuing education work, and continuous	1.Lead the discipline development of specialized nursing team<br>2.Serving as lecturer of school specialty courses or invited as a nursing expert to be the host/reporter of professional conferences/seminars<br>3.Organize and plan specialty competitions and act as an examiner<br>4.Organize and plan group health-related service activities (clinic, health consultation, etc.).<br>5.Assist relevant departments to establish training bases for specialized nurses and

		activities	departments.<br>5.Participate in group health-related service activities (clinic, health consultation, etc.)<br>6.Participate in nursing academic exchange activities	tracking and improvement.<br>5.To undertake the hospital specialty education courses, preside over the hospital specialty knowledge and skills examination<br>6.Guide lower level specialist nurses to carry out specialty health education activities and continuous improvement<br>7.Organize and plan group health-related service activities (clinic, health consultation, etc.).<br>8.Participate in nursing academic exchange activities	develop training programs
**Leadership and management skills**	1.Specialty basic goods, equipment, drug supplies management<br>2.Specialty group paperwork organization	1.Real-time evaluation of the quality of specialty care by lower-level specialist nurses, and timely correction of inappropriate nursing	1.Participate in specialty care quality continuous improvement program<br>2.Serving as a member of the hospital-level specialty care quality	1.Acting as the head/vice head of the hospital-level specialty group<br>2.As the departmental specialty care quality control team leader, is	1.The development of specialty care development plan and organization and implementation, management of specialty

		behavior	control team / departmental specialty care quality control team leader<br>3.Analysis of the implementation of the opinions of specialty care consultation and improvement<br>4.Analysis of patient's satisfaction with specialty care work and improvement	responsible for completing the specialty quality control briefings, and summarize the problems in specialty care, continuous improvement to improve the quality of specialty care<br>3.Can evaluate the current specialty care system, routine and emergency plan, and put forward proposals for amendment or updating.<br>4.Chairman of specialty nursing quality continuous improvement program<br>5.Assist the specialty team leader to be responsible for the comprehensive assessment and evaluation of NP1, 2 and 3 level specialist nurses.	care work<br>2.Serve as the head of the hospital-level specialty team.<br>3.As the hospital-level specialty care quality control team leader, responsible for completing the specialty quality control briefings, and summarize the problems in specialty care, and continuously improve the quality of specialty care.<br>4.Developing and revising specialized nursing system, routine and emergency plan, etc.<br>5.Participate in the comprehensive assessment and evaluation of nursing staff organized by the Nursing Department.<br>6.Responsible for the comprehensive assessment and evaluation of

					specialized nurses at NP1, 2 and 3 levels.<br>7.As a member of national, provincial and municipal specialty societies and other social organizations.
**Clinical research capacity**	1.Actively consult relevant literature at home and abroad, and understand the cutting-edge dynamics of the specialty, etc.<br>2.Engage in basic nursing research under the guidance of higher level specialized nurses.<br>3.Write nursing cases and papers	1.Actively consult relevant literature at home and abroad, and understand the cutting-edge dynamics of the specialty, etc.<br>2.Engage in basic nursing research under the guidance of higher level specialized nurses.<br>3.Write cases and papers<br>4.Submit papers and abstracts for specialty-related conferences.	1.Responsible for specialty nursing education in the department, and undertake part of the hospital level specialty nursing education work<br>2.Submit papers, abstracts, etc. for specialty-related conferences and accepted for poster/oral communication (first or corresponding author only).<br>3.Publish papers in provincial and above journals as the first/corresponding author.	1.Ability to identify problems in nursing practice in order to conduct nursing research<br>2.Participate in nursing research<br>3.Publish scientific and technological papers as the first/corresponding author/report patents and achievements.<br>4.Translate evidence and apply nursing research results under the guidance of senior specialist nurses.<br>5.Submission of specialty-related conference papers,	1.Developing and guiding the implementation of expert consensus, guidelines, protocols, etc. in specialized areas<br>2.To develop new business, new technologies and new methods of specialized nursing care<br>3.Publishing scientific and technological papers as the first/corresponding author/reporting patents/participating in the writing of treatises, etc.<br>4.Obtaining provincial or above scientific research awards

			4.Mentor junior specialized nurses to participate in nursing research and paper writing<br>5.Participate in nursing research<br>6.Winning research awards at the hospital level.	abstracts, etc., and accepted oral communication (limited to the first or corresponding author).<br>6.Obtaining municipal and above all kinds of scientific research awards.	5.declaration and organization of specialized nursing academic exchange activities<br>6.Acting as the host/co-host of the research project<br>7.Specialty related conference papers, abstracts, etc., and accepted oral communication (limited to the first or corresponding author)

Note: Complex cases are adjudicated by the specialty team leader.

四、Continuing Education

Compulsory courses at the nurse specialist level

	NP1	NP2	NP3	NP4	NP5
**Annual Assessment Course**	1.Specialty knowledge and skill training at hospital level<br>2.Specialty related provincial continuing education programs<br>3.Difficult cases	1.Specialty knowledge and skill training at hospital level<br>2.Specialty related provincial continuing education programs<br>3.Difficult cases	1.Specialty knowledge and skill training at hospital level<br>2.Specialty related provincial continuing education programs<br>3.Difficult cases	1.Specialty knowledge and skill training at hospital level<br>2.Specialty related provincial continuing education programs<br>3.Difficult cases	1.Specialty knowledge and skill training at hospital level<br>2.Specialty related provincial continuing education programs<br>3.Difficult cases

		discussion<br>4.Nursing literature sharing salon	discussion<br>4.Nursing literature sharing salon	discussion<br>4.Nursing literature sharing salon	discussion<br>4.Nursing literature sharing salon	discussion<br>4.Nursing literature sharing salon
**Required Courses for Advancement**	**Ability to provide direct clinical care**	1.Annual development plan for specialized nurses<br>2.Specialty nursing development system<br>3.Work summary and plan writing skills	Annual Development Plan for specialist nurses	Annual Development Plan for specialist nurses	Annual Development Plan for specialist nurses	Annual Development Plan for specialist nurses
	**Capacity for ethical decision-making**	1. Nursing ethics and law	1.Ethical issues in clinical work	1.Nursing Ethical Decision Making Theory and Methods	1.Notes on Ethical Applications for Clinical Trials	1.Clinical Practice of a Shared Patient-Care Decision-Making Model
	**Provision of advisory capacity**	1.Cultivation of critical thinking ability<br>2.Application of critical thinking in clinical care<br>3.Specialty difficult case discussion norms<br>4.Consultant Role of specialist nurses	1.Consultation process and standardization<br>2.Establishment and implementation of consultation mode<br>3.Multidisciplinary consultation mode application in clinical practice	1.Establishment and Practice of Specialized Nursing Clinics	1.Construction and application of a specialized classic case base	Establishment and practice of out-of-hospital specialized nursing workstations
	**Expert guidance capacity**	1.Common teaching theories, methods and techniques	1.Analysis and evaluation of teaching needs<br>2.Common Teaching	1.Specialty curriculum system design<br>2.Clinical teaching	1.Construction and training of specialized nursing faculties	1.Sharing of fine teaching cases<br>2.Discipline

	2.How to teach a good lesson<br>3.Bedside Teaching Skills<br>4.Retrieval and application of network teaching resources<br>5.Health education mode and program	Tools and Means<br>3.Design and production of teaching media<br>4.Organization and implementation of clinical teaching<br>5.Design and implementation of health education	management and quality control		development and design
**Ability to work with others**	1.Interpersonal Communication and Emotion Management for Nurses	1. Communication dilemmas in clinical practice	1.Communication and cooperation in multidisciplinary teams	1.Clinical practice of a nurse specialist-led multidisciplinary collaboration model	1. Establishment and management of a multidisciplinary cooperation model
**Leadership and management skills**	1.Common theories and methods of nursing management<br>2.The importance of nursing quality control in nursing management<br>3.Application of nursing quality continuous improvement methods and tools	1.Nursing quality continuous improvement case sharing<br>2.Nursing quality control brief writing skills	1.Nursing Leadership Cultivation and Development<br>2.Nursing human resource management	1.Nursing risk prevention and control<br>2.Nursing care quality sensitive indicators monitoring and quality improvement	1.Specialized nursing quality index system construction<br>2.Precision nursing quality management
**Clinical research capacity**	1.Literature search and evaluation<br>2.Learning of common	1.Commonly used statistical methods<br>2.Innovative thinking and	1.Evidence-based nursing series of courses<br>2.Tips for writing an	1.SCI Series Courses<br>2.Research Selection and Bid Writing	1.Scientific research project declaration skills

	scientific research methods (RCT\CCT\survey type, qualitative research)<br>3.Application of Literature Management Software<br>4.Case management methods and case report writing	patent application<br>3.Writing and Submission of Scientific Papers<br>4.Abstract writing and submission of conference abstracts	opening report		2.Science and technology awards declaration points

Note: 1. Annual assessment courses refer to the courses that require the completion of study and assessment every year;
2. Courses required for promotion refer to courses that require completion of study and assessment during the period of service.

五、Appraisal standard

Specialized nurses will be assessed once a year. The head and deputy head of the specialized team and the Nursing Department will conduct the assessment in accordance with the assessment standards based on the performance of specialized nurses' post duties in each tier every year, and will be responsible for the authenticity of the assessment results. (Completion of continuing education courses within the corresponding tier during the assessment period)

		NP1	NP2	NP3	NP4	NP5
**Core competencies**	**Ability to provide direct clinical care**	1.Pass the annual assessment of the corresponding level of nurses<br>2.Complete at least 3 cases of specialized	1.Pass the annual assessment of the corresponding level of nurses<br>2.Complete at least 3 cases of specialized	1.Pass the annual assessment of corresponding level nurses<br>2.Independently complete at least 3 cases of	1.through the N3 level nurse annual assessment<br>2.Independently complete at least 5 cases of specialized complex case care	1.Pass the annual assessment of N3 level nurses<br>2.Specialized nurses at the lower level are instructed to complete

		nursing cases under the guidance of higher level specialized nurses.<br>3.Participate in no less than 80% of specialized knowledge and skill training at the hospital level.<br>4.Completion of 5 points of Class I credits of specialty-related provincial continuing education programs.<br>5.Complete the annual work summary 1<br>6.Complete the annual plan of personal development 1	complex case nursing under the guidance of superior specialized nurses<br>3.Independently complete at least 5 cases of specialized nursing care.<br>4.Participate in at least 80% of specialized knowledge and skills training at the hospital level.<br>5.Complete the specialty-related provincial continuing education program with 10 points of class I credits.<br>6.Complete the annual work summary 1<br>7.Complete the annual plan of personal development 1	specialized complex case care<br>3.Participate in at least 80% of specialized knowledge and skills training at the hospital level.<br>4.Finish 10 credits of provincial continuing education program related to the specialty.<br>5.Complete an annual work summary<br>6 、 Complete 1 annual personal development plan	3.Participate in at least 80% of specialized knowledge and skill training at the hospital level.<br>4.Complete 15 points of provincial continuing education programs related to the specialty.<br>5.Complete an annual work summary<br>6.Complete 1 annual personal development plan	at least 10 cases of specialized complex case care.<br>3.Participate in at least 80% of specialized knowledge and skills training at the hospital level.<br>4.Completing 15 points of provincial continuing education programs related to the specialty.<br>5.Finish 1 annual work summary<br>6.To complete the personal development of the annual plan 1
	**Capacity for ethical**	1.Obtaining informed consent from patients	1.Obtain informed consent from patients	1.Obtain informed consent from patients and	1.Obtain informed consent from patients and	1.Obtain informed consent from patients

	**decision-making**	and their families before implementing specialty care	and their families before performing specialty care.<br>2.Can follow the ethical principles to develop a specialized care program, to protect the basic rights and interests of patients	their families before performing specialty care.<br>2.Can follow the ethical principles to develop a specialized care program, to protect the basic rights and interests of patients	their families before performing specialty care.<br>2.Can follow the ethical principles to develop a specialized care program, to protect the basic rights and interests of patients	and their families before implementing specialized nursing care.<br>2.Can follow the ethical principles to develop specialized care programs to protect the basic rights and interests of patients
	**Provision of advisory capacity**	1.Participate in the discussion of at least 5 cases of specialized difficult cases at the hospital level and within the specialty group	1.Participate in at least 5 cases of specialized difficult case discussions at the hospital level and within the specialist group.<br>2.Participate in at least 2 cases of specialty consultation under the guidance of higher level specialist nurses.	1.Participate in at least 5 difficult case discussions at the hospital level and within the specialty group.<br>2.Carry out at least 2 cases of specialty consultation.	1.Chairing at least 5 cases of difficult case discussions within the specialty group<br>2. To carry out at least 5 cases of specialty consultation	1.Chairing the discussion of at least 5 difficult specialized cases in the specialty group.<br>2.Provide consultation and consultation on difficult nursing cases within and outside the hospital for at least 10 times.
	**Expert guidance capacity**	1. Participate in at least 2 group health-related service activities (clinics,	1.Lead and teach at least 1 lower level specialized nurse<br>2.As a lecturer to	1.Lead and teach lower level specialized nurses, advanced training nurses and specialized nurse	1.the development of annual hospital-level specialty training program 1	1.Leading and teaching subordinate specialized nurses, advanced training nurses and

		health counseling, etc.)	undertake hospital / departmental specialty training at least 3 times (must provide lesson plans, PPT, sign-in sheet and proof of photos)<br>3、Participate in at least 2 group health-related service activities (clinic, health consultation, etc.) as a lecturer/specialist (activity planning plan/summary, supporting photos)	training course students 1-2 people<br>2.Organize and plan at least 3 hospital-level/departmental specialty trainings (lesson plans, PPTs, sign-in sheets and supporting photos must be provided)<br>3.Participate in at least 1 group health-related service activity (clinic, health consultation, etc.) as a lecturer/specialist (activity planning plan/summary, supporting photos)<br>4、Organize and plan at least 1 hospital/departmental specialist health education activity (supporting documents: health education program, summary and	2.Teaching lower level specialized nurses, advanced training nurses and specialized nurse training course students 2-3 people<br>3.Organize and plan at least 1 hospital/departmental specialty training (lesson plan, PPT, sign-in sheet and supporting photos must be provided)<br>4.Participate in at least 1 group health-related service activity (clinic, health consultation, etc.) as a lecturer/specialist (activity planning plan/summary, supporting photos)<br>5.Organize and plan at least 1 out-of-hospital, internal/departmental specialist health education activity	specialized nurses training course students 3-4 people<br>2.Organize, plan and carry out specialty competitions and serve as an examiner at least once<br>3.Participate in at least 1 group health-related service activity (clinic, health consultation, etc.) as a lecturer/expert (activity planning plan/summary, supporting photos)<br>4.Organize and plan at least 1 out-of-hospital and in-specialty health education activity (supporting documents: health education program, summary and photos)<br>5.As a school professional course

				photos)	(supporting documents: health education program, summary with photos)<br>6.Serving as a lecturer in school professional courses or invited as a nursing expert to be a host/reporter of a professional conference/seminar at least 1 time	lecturer or as a nursing expert invited to serve as a professional conference / seminar presenter / reporter at least 1 time<br>6Chairing 1 national, provincial or municipal continuing education training course<br>7.To assist the relevant departments to establish a specialized nurse training base and develop training programs
	**Ability to work with others**	1.Able to coordinate and cooperate effectively with other members in the specialized team (no less than 70 points in the evaluation of team members)	1.Be able to coordinate and work effectively with other members in the specialist group (with a rating of not less than 80 points from the members of the group)	1.Be able to coordinate and work effectively with other members within the specialty team (with a team member rating of at least 80 points)<br>2.Provide at least 3 records of participation in multidisciplinary team consultations	1.Ability to coordinate and work effectively with other members within the specialty team (with a team member rating of at least 80 points)<br>2.Provide at least 5 records of hosting multidisciplinary team consultations	1.Ability to coordinate and work effectively with other members within the specialty team (with a team member rating of at least 80 points)<br>2.Provide at least 10 records of hosting multidisciplinary team

					3.Provide at least 2 records of participating in joint medical and nursing room visits within the department	consultations<br>3.Provide at least 2 records of participating in hospital-level joint medical and nursing room visits
	**Leadership and management skills**	1.Responsible for the management of specialized basic items, equipment, drug supplies<br>2.Responsible for the organization of specialized group instruments<br>3.Specialty nursing quality control team members<br>4.Participate in at least 1 continuous improvement project of specialty nursing quality	1.As a member of the hospital-level specialty care quality control team<br>2.Participate in at least 1 continuous improvement project of specialty nursing quality.	1.As a member of the hospital-level specialty care quality control team / departmental specialty care quality control team leader<br>2.Chairing at least 1 project for continuous improvement of specialty care quality.<br>3.As a departmental specialty nursing quality control team leader to complete the annual, quarterly and monthly specialty quality control briefings	1.Serving as the hospital-level specialty group leader / deputy group leader<br>2.Preside over at least one specialty nursing quality continuous improvement project or the annual specialty indicators to achieve the standard rate of 100%.<br>3.As the hospital-level specialty quality control team leader / deputy team leader, the team leader needs to complete the annual, quarterly and monthly specialty quality control briefings<br>4.The development of	1.Serving as a hospital-level specialty team leader<br>2.Chairing at least one continuous improvement project of specialty nursing quality or achieving 100% of the annual specialty indicators.<br>3.Acting as the head of the hospital-level specialty nursing quality control team, and completing the annual and quarterly hospital-level specialty quality control briefings.<br>4.Regularly revise

					emergency response plan for specialized emergencies, and hosted at least one emergency drill<br>5.Develop the job duties and procedures of specialized nurses, and continuously improve them.<br>6.Participate in the comprehensive assessment of NP1, 2 and 3 level specialized nurses.	specialty nursing system, routine and emergency plan, etc.<br>5.Responsible for the comprehensive assessment of specialized nurses at NP1, 2 and 3 levels.<br>6.National, provincial, municipal societies and other social positions
	**Clinical research capacity**	1.Complete a total of 1 or more annual case reports.<br>2.Participate in nursing literature sharing salon≥3 times	1.Complete a total of 1 or more annual case reports.<br>2.Submit at least 1 paper, abstract, etc. for specialty-related conferences<br>3.Participate in nursing literature sharing salon ≥3 times	1.Complete 1 or more annual case reports.<br>2.Publish 1 paper in provincial or above periodicals as the first author or corresponding author or get 1 utility model patent approved.<br>3.Accepted conference poster/oral communication (limited	1.Completing 1 or more annual case reports.<br>2.As the first author or corresponding author to publish a scientific and technological core and above journals or approved invention patents 1<br>3.Accepted conference oral communication	1.Completion of 1 or more annual case reports in total<br>2.As the first author or corresponding author to publish a scientific and technological core and above journal papers or approved invention patents 1<br>3.Accepted conference

				to the first or corresponding author)<br>4.Participate in at least one project within three years<br>5.Obtain at least 1 research award at the hospital level.<br>6.Participate in nursing literature sharing salon ≥ 3 times	(limited to the first or corresponding author)<br>4.Participate in at least one project within three years<br>5.Obtaining at least 1 research award at municipal level or above<br>6.Participate in nursing literature sharing salon ≥ 3 times	oral communication (limited to the first or corresponding author)<br>4.Within three years to preside over/co-preside over at least 1 project<br>5.Acquired at least 1 provincial and above research awards<br>6.Participate in nursing literature sharing salon ≥ 3 times<br>7.The organization of specialist groups to carry out specialist nursing new business, new technologies / appropriate technology at least 1

Note: Complex and difficult cases are determined by the specialty team leader.

六、Criteria for recognizing assessment results

1.NP1, 2, 3 tier specialized nurses by the specialty NP5, 4 nurses to carry out comprehensive assessment; if the specialty does not have NP5, 4 nurses by the specialty team leader and deputy team leader to carry out comprehensive assessment; team leader and deputy team leader by the Department of Nursing unified comprehensive assessment;

2.NP4, 5 level specialist nurses to participate in the Department of Nursing organized by the comprehensive assessment of specialist nurses;

3.The assessment results are determined as qualified, basic qualified and unqualified according to the assessment scores: if the assessment scores are ≥80, the annual assessment will be designated as qualified; if the assessment scores are 60≤80, the annual assessment will be designated as basic qualified; if the assessment scores are <60, the annual assessment will be designated as unqualified; and those who are in serious violation of discipline or serious nursing adverse events (I and II) will be directly designated as unqualified.

七、Position management procedures for specialized nurses

1.Initial grading, promotion and further training of specialized nurses should be submitted by individuals, and then submitted to the Nursing Department for examination and decision after the recommendation of the department.

2.All the initial classification of specialized nurses will be classified as NP1 in the first year, and participate in the training and examination of NP1 level. The second year is the period of classification and assessment. During the period of classification and assessment, according to the requirements of each level, they should participate in the training and assessment of the level they are qualified for. At the end of the one-year assessment period, if they meet the requirements of the level, they will be graded according to that level, and thereafter all of them will be promoted according to the promotion standards in these Regulations.

3.The title and academic qualifications of specialized nurses shall be based on the certificates obtained.

4.The number and proportion of specialized nurses at each level in different specialties shall be reviewed and decided by the Nursing Department.

5.After the annual assessment of the specialty group is completed, the assessment form will be submitted to the Nursing Department for review, and I will sign and confirm the form for filing, and it will be used as the basis for the promotion in the next year: those who fail in the annual assessment/three years with two basic passes/consecutively with two basic passes in the assessment are downgraded to the first level; those who fail in the three years with two failures/consecutively with two failures in the assessment will lose their qualification of specialist nurses, and they will be adjusted to corresponding N level positions for management by the specialty group after submitting the form to the Nursing Department. The specialty team will report to the nursing department and adjust to the corresponding N-level position for management.

6.due to personnel transfer and other circumstances caused by the change of direction of the specialist nurse, the department reported to the Department of Nursing to discuss the decision. (Whether the management of specialist liaison nurses is included in the management of specialist nurses)
